# An Overview of Fotemustine in High-Grade Gliomas: From Single Agent to Association with Bevacizumab

**DOI:** 10.1155/2014/698542

**Published:** 2014-03-31

**Authors:** Giuseppe Lombardi, Patrizia Farina, Alessandro Della Puppa, Diego Cecchin, Ardi Pambuku, Luisa Bellu, Vittorina Zagonel

**Affiliations:** ^1^Medical Oncology 1 Unit, Venetian Oncology Institute-IRCCS, Via Gattamelata 64, 35128 Padua, Italy; ^2^Neurosurgery Department, Padua Hospital, Padua, Italy; ^3^Nuclear Medicine Service, Department of Diagnostic Medical Sciences, University of Padua, Padua, Italy

## Abstract

Fotemustine is a third-generation nitrosourea showing efficacy in various types of tumors such as melanoma and glioma. We reviewed the most important studies on fotemustine treatment in glioma patients analyzing its pharmacological profile and its activity and safety. Fotemustine was used as single agent or in association with new targeted drugs such as bevacizumab; fotemustine was used both as first-line chemotherapy before temozolomide era and in refractory-temozolomide patients during temozolomide era. Finally, analyzing and comparing the activity and safety of fotemustine alone or in combination with bevacizumab versus other nitrosoureas such as lomustine, we may suggest that the combination treatment with bevacizumab and fotemustine may be active and tolerable in patients with high grade gliomas.

## 1. Introduction

Fotemustine is a third-generation nitrosourea showing efficacy in melanoma, some haematological tumors, and gliomas. Malignant gliomas account for approximately 50% of all malignant primary brain tumors in adults; high-grade gliomas include anaplastic astrocytomas, anaplastic oligodendrogliomas, mixed anaplastic oligoastrocytomas, anaplastic ependymomas, and glioblastomas. Standard therapy for newly diagnosed malignant glioma includes surgical resection when feasible, radiotherapy, and chemotherapy. Presently, there is no consensus therapy recommended for the treatment of recurrent malignant gliomas; a second debulking surgery could be proposed as well as a second course of chemotherapy. However, despite optimal treatment, median survival ranges from 12 to 15 months for glioblastoma and from 2 to 5 years for anaplastic gliomas [1]. Various antineoplastic agents such as temozolomide, procarbazine, carmustine, lomustine, and vincristine, or some combinations of those, were used. Numerous phase II studies showed an important activity of fotemustine in high-grade gliomas, especially in glioblastoma, as first-line treatment or in recurrent disease [2].

Fotemustine (diethyl 1-{1-[3-(2-chloroethyl)-3-nitrosoureido] ethyl} phosphonate is an alkylating cytotoxic agent, belonging to the group of nitrosourea. It is characterized by elevated lipophilic properties and a low molecular weight that contribute to facilitate its passage through the blood-brain barrier [3]. Moreover, fotemustine shows an important diffusion in neuronal cells and glia. The antitumor activity of fotemustine is related to its ability to alkylate DNA. In particular, fotemustine decomposes quickly in aqueous solution giving rise to two principal compounds that produce a cytotoxic damage through a mechanism based on alkylating process. The most important step is the alkylation of DNA O^6^-guanine followed by DNA two-strand cross-linking events. Therefore, the presence of high-levels of O^6^-methylguanine-DNA-methyltransferase enzyme (MGMT), an enzyme able to remove alkyl adducts from the O^6^ position of guanine, confers resistance to fotemustine. Methylation of the MGMT promoter results in gene inactivation, thus potentially leading to increased sensitivity to treatment. After intravenous infusion, the plasma concentration reached the steady-state in 45 minutes and the plasma concentration varied between 1 and 14 ug/mL, disappearing in the blood within three hours. Fotemustine concentration in the cerebrospinal fluid reached 23% of plasma level. Its excretion is mainly urinary and fecal excretion is minimal [4]. Regarding its adverse events, the most important toxic events are thrombocytopenia, leukopenia, and anemia, while liver and kidney toxicity are moderate [5].

The combination of procarbazine, lomustine, and vincristine is the first-line treatment for anaplastic oligodendroglioma [6]; temozolomide is the gold standard first-line treatment of glioblastoma [7] and after its introduction fotemustine has been used as a treatment for recurrent disease. However, fotemustine have been used as single or in-combination agent, during radiation treatment and in association with new targeted drugs such as bevacizumab. And so, in this review, we describe the different uses and combinations of fotemustine in clinical practice for glioma patients, before and during temozolomide era.

## 2. Methods

We performed a systematic literature search in January 2014. Studies on the use of fotemustine for the treatment of malignant gliomas were identified by searching the Medline electronic database (1990–2014). Individual conference proceedings from the ASCO (American Society of Clinical Oncology) Annual Meeting (2009–2013) were searched on their online interface. The search strategy included terms used to describe malignant gliomas, fotemustine and temozolomide, and antiangiogenic treatment. For further relevant studies this search was supplemented by reviewing the bibliographies of key papers.

## 3. Fotemustine before Temozolomide Era

In 1991, Frenay et al. [8] performed a phase II study of fotemustine in recurrent supratentorial malignant gliomas (see Table 1); they analyzed 38 patients including glioblastomas multiforme, anaplastic astrocytomas, and pineoblastomas. Fotemustine was given intravenously at the dose of 100 mg/m^2^ infused over 1 h. The treatment plan consisted of one administration weekly, for 3 consecutive weeks (induction treatment), followed by a 5-week rest period. Fotemustine was then given every 3 weeks (maintenance treatment) until toxicity or progressive disease. Prior treatment included surgical resection (extensive and partial) in 71% of patients, radiotherapy in all patients, and chemotherapy in 26% of cases. All patients received the induction treatment, but only 13 received the maintenance treatment. Overall, 26% of patients responded to treatment (partial response), for a disease control rate (DCR) (responses plus stabilizations) of 73%. In patients with partial response, the median progression free survival (PFS) was 32.7 weeks and the median overall survival (OS) from fotemustine treatment was 40 weeks, while in patients with stable disease PFS and OS were 21 and 42.5 weeks, respectively; in 10 patients who failed to respond the OS was 15.2 weeks. The most common side effect was a delayed reversible and cumulative haematological toxicity: thrombocytopenia and leukopenia grade 3-4 toxicities were observed in 23% and 17.2% of patients, respectively. Moreover, prior chemotherapy was associated with more frequent haematological toxicity.

Ozkan et al. [9] analyzed the role of fotemustine after surgery and radiotherapy in high-grade gliomas. After surgery and radiotherapy, fotemustine was given at a dose of 100 mg/m^2^ every 3 weeks for 6 cycles in 27 patients. All of the patients completed their radiotherapy (60 Gy in 30 fractions) within the predetermined 6-week period; 89% of the patients had a ECOG-PS 0-1 and 11% ECOG PS 2. The median PFS and OS were 11 and 8 months, respectively. Grade 3 neutropenia was the most important toxicity (7% of patients).

In another study, Fazeny-Dörner et al. [10] assessed the activity and toxicity of a combination of dacarbazine and fotemustine in 31 nitrosourea-pretreated patients with recurrent glioblastoma multiforme. Previously, lomustine monotherapy or lomustine-combination therapy was administered to all patients. PFS and OS were 17 and 45 weeks, respectively. Disease control rate was 55%. The most important toxicity was grades 3-4 thrombocytopenia (10%).

Boiardi et al. [11] performed a phase I study to determine the maximum tolerated dose (MTD) and toxicity profile of fotemustine when combined with procarbazine. Sixteen patients received an induction cycle consisting of 100 mg/day oral procarbazine for 12 consecutive days and a 1 h infusion of fotemustine given 4 hours after procarbazine on days 5 and 12 at escalated doses. After a 6-week rest period, a maximum of 4 maintenance cycles (procarbazine 300 mg/day, 4 days; fotemustine, day 4) were given every 4 weeks. 15 (94%) patients performed a presurgery and a prenitrosourea chemotherapy; all patients had a preradiation therapy. The MTD of fotemustine was 125 mg/m^2^ (days 5 and 12); at this dose level, 33% of patients had grade 3 anemia, 17% grade 3 leucopenia, and 17% grade 3 thrombocytopenia. Overall, they obtained a DCR of 50%. Median PFS and OS were 2.6 and 9.7 months, respectively.

An interesting study was performed by Frenay et al. [12]; they analyzed the activity and toxicity of combination of fotemustine (100 mg/m^2^, day 1), cisplatin (33 mg/m^2^, days 1–3), and etoposide (75 mg/m^2^, days 1–3), repeated monthly, in 33 nonoperable glioblastoma patients. Radiation therapy was initiated if a patient had a progression disease during the chemotherapy. They reported a DCR of 79% with 1 complete response. The median OS was 10 months. 36% of patients had grades 3-4 haematological toxicity.

## 4. Fotemustine during Temozolomide Era

After the introduction of temozolomide, the standard first-line therapy for glioblastoma patients was radiation therapy in combination with temozolomide followed by six cycles of temozolomide [7]. Regarding second-line treatment in patients with recurrent glioblastoma there is no standard therapy. In the last years, there was interest in the role of bevacizumab, alone or in combination to cytotoxic drugs but the results were conflicting [13–16]. And so, in this contest, fotemustine was tested always in temozolomide-refractory patients and can be a valid second-line treatment (see Table 2).

Scoccianti et al. [17] evaluated the activity of fotemustine in 27 patients with progressive or recurrent glioblastoma after failure of postoperative treatment with radiation therapy plus temozolomide. They obtained a median PFS and OS of 5.7 months and 9.1 months, respectively with a disease response rate of 48%. Grade 3 thrombocytopenia developed in 11% of patients while 4% of patients had grade 4 leukopenia.

Brandes et al. [18] studied the activity and toxicity of fotemustine in 43 patients with recurrent glioblastoma. All patients received fotemustine as second-line treatment. All patients received three-weekly doses (100–75 mg/m^2^) of fotemustine (induction phase) followed, after a 5-week rest, by fotemustine (100 mg/m^2^) every 3 weeks (maintenance phase). Disease control rate was 42.5% (3 partial responses and 15 stable diseases) and that was significantly greater in methylated than unmethylated MGMT patients, 75 and 34.6% (*P* = 0.044), respectively. Median PFS was 1.7 months and PFS-6 in the population treated was 22.5%; moreover, patients that initiated fotemustine at least 3 months after temozolomide completion showed a significantly higher PFS-6 (30.7 versus 16.7%) than patients who initiated fotemustine immediately after temozolomide completion (*P* = 0.034). Median OS was 6 months and there was no statistical difference between methylated and unmethylated MGMT promoter status. Grades 3 and 4 thrombocytopenia and neutropenia were observed in 21% and 16% of patients, during the induction phase and in 0 and 9.5% patients during the maintenance phase, respectively.

A similar study was conducted by Fabrini et al. [19] using the conventional schedule of fotemustine. They analyzed 50 patients with recurrent glioblastoma obtaining a median PFS and OS of 6.1 and 8.1 months, respectively; the disease control rate was 62% (1 complete response, 8 partial responses, and 22 stable diseases). Grade 3 thrombocytopenia was documented in 8% of patients, 2% of patients developed grades 3-4 neutropenia, grade 3 anaemia, and grade 3 lymphopenia.

Subsequently, Addeo et al. [20], in a prospective phase II study, tested a new schedule of fotemustine in temozolomide-pretreated patients with glioblastoma; all patients underwent fotemustine 80 mg/m^2^ every 2 weeks for five consecutive administrations (induction phase) and then every 4 weeks at 80 mg/m^2^ as maintenance. This schedule was generally well tolerated with a good efficacy; in fact, the median PFS and OS were 6.7 and 11 months, respectively; no significant differences were found between median PFS in relation to KPS (Karnofsky Performance Status), age, and status of MGMT promoter. Moreover, only 7% and 3% of patients developed grade 3 thrombocytopenia and leukopenia, respectively.

Fabi et al. [21] conducted a phase II study to address whether administration of fotemustine at 60 mg/m^2^ weekly for 3 cycles followed, after a 5-week rest period, by fotemustine 75 mg/m^2^ every 3 weeks would preserve clinical activity with the advantage of improved tolerance. They analyzed 40 patients with recurrent malignant glioma: 30 glioblastomas, 6 anaplastic astrocytomas, and 4 anaplastic oligodendrogliomas. Previously, all patients underwent temozolomide therapy. The median PFS and OS were 3 and 6 months, respectively. Methylated MGMT patients appeared to experience both a longer PFS and OS, although this did not reach statistical significance. All patients were evaluable for toxicity: 7% of patients developed grade 3 thrombocytopenia, 10% of patients developed grade 3 leukopenia, and about 2% of patients developed grade 3 hypertransaminasemia or nausea/vomiting.

Santoni et al. [22] performed an interesting study analyzing retrospectively the activity and toxicity of second-line fotemustine in elderly patients with recurrent glioblastoma. They obtained good results with a median PFS and OS of 4.2 and 7.1 months, respectively; the disease control rate was 43% with 1 complete response, 12 partial responses, and 18 stable diseases. The most relevant grades 3-4 toxicity events were thrombocytopenia (15%) and leukopenia (9%). Noteworthy, in the multivariate analysis, the time from radiation therapy and the number of temozolomide cycles resulted as prognostic factors.

In another study, Silvani et al. [23] analyzed the activity and safety of procarbazine and fotemustine combination in the treatment of temozolomide-refractory glioblastoma patients. Noteworthy, they obtained a low rate of toxicity: only 2% of patients had grades 3-4 haematologic toxicity and no patients required dose reduction. On the other side, they demonstrated interesting results about efficacy: median PFS and OS were 19.3 and 28.7 weeks, respectively.

The association of temozolomide and fotemustine in temozolomide-refractory glioblastoma was unsuccessful in two studies [24, 25] due to the important toxicity of the combination; in fact, the study of Gaviani et al. [25] was stopped for relevant side-effects that occurred in the first 20 patients: 13 patients did not complete the third cycle because all the patients experienced grades 3-4 thrombocytopenia and 7 of 13 patients had grade 4 granulocytopenia.

## 5. Fotemustine in Association with Bevacizumab: Is Fotemustine the Appropriate Cytotoxic Drug for the Combination Therapy?

High grade gliomas are highly vascularised brain tumors and are therefore attractive targets for antiangiogenic therapies. Vascular endothelial growth factor (VEGF) is an important regulator of angiogenesis and invasion and is highly expressed within brain tumors. Bevacizumab, an anti-VEGF antibody, was approved by the US Food and Drug Administration in 2009 for patients with recurrent glioblastomas who have failed previous temozolomide and radiation therapy based on prior studies [16, 26]. Moreover, a recent clinical trial showed encouraging evidence of bevacizumab activity as well as acceptable safety among patients with recurrent grade III malignant glioma [27]. It has been demonstrated that during bevacizumab therapy tumors may evade the inhibition of VEGF signaling and so the association of a cytotoxic drug might lead to more effective treatment. On the other hand, bevacizumab might enhance the delivery of an active cytotoxic drug.

In a very interesting study, Soffietti et al. [28] performed a prospective phase II study analyzing the combination of bevacizumab and fotemustine for recurrent glioblastoma patients (see Table 2). Fifty-four patients with recurrent glioblastoma were enrolled and were treated with fotemustine 75 mg/m^2^ on days 1 and 8, bevacizumab 10 mg/Kg on days 1 and 15 followed, after a 3 week-rest period, by fotemustine 75 mg/m^2^ plus bevacizumab 10 mg/Kg on day 1 every 3 weeks; the median age of the patients was 57.1 years, 44% of patients had a KPS of ≥90, 85% had unifocal tumors, and 15% of patients had multifocal tumors. PFS rate at 6 months (PFS-6) was 42.6% and the median PFS was 5.2 months. MGMT gene status was associated with PFS, although not statistically significant in the adjusted analysis. OS rate was 75.9% and 29.7% at 6 and 12 months, respectively, and the median OS was 9.1 months. The risk of death increased in patients with the worst performance status while, second surgery, MGMT gene status and tumor extension did not influence OS. Noteworthy, the disease control rate was very high: 89% of patients had disease control with 4% of complete responses, 48% of partial responses, and 37% of stable diseases; only 11% of patients had a progression disease. Response was not predicted by any clinical factor, including the MGMT gene status. Grade 3 toxicities were predominantly haematologic, including neutropenia (13%) and thrombocytopenia (9%); toxicities due to bevacizumab were deep venous thrombosis (4%), pulmonary embolism (4%), and hypertension (2%); however, 22% of patients with persistent grade 3 or 4 neutropenia or thrombocytopenia discontinued fotemustine or required dose modification. Likely, employing a fotemustine schedule with a lower toxicity, more patients could continue the treatment, for example, the fotemustine schedule used by Addeo et al. [20] (see Table 2) which reported a very low toxicity.

In another study, the same treatment was performed in grade III gliomas [29]. Thirty-two patients with grade III gliomas recurrent after surgery, radiation therapy, and concomitant/adjuvant temozolomide were enrolled in a phase II prospective study. The median age was 46 years, the median PFS was 5 months, and the median OS was 8.6 months. Disease response rate was 94%. Five patients (16%) interrupted the treatment for grade III and IV myelotoxicity.

In a recent Dutch phase II randomized study on recurrent glioblastoma (BELOB trial) [30] presented at American Society of Clinical Oncology (ASCO) 2013 Congress patients were assigned to either bevacizumab 10 mg/Kg every 2 weeks, bevacizumab 10 mg/KG every 2 weeks, and 110 mg/m^2^ lomustine every 6 weeks, or lomustine 110 mg/m^2^ every 6 weeks. Primary endpoint was 9 months of overall survival (OS-9). Overall, 148 patients were considered eligible, median age was 57 years (range 22–77), and median WHO Performance Status was 1. Noteworthy, the dosage of lomustine in the combination arm was lowered to 90 mg/m^2^ because of haematological toxicity (predominantly thrombocytopenia), and at this lower lomustine dose-level the combination treatment was in general well tolerated. The OS-9 was 59% in the combination treatment and 38% with bevacizumab alone and 43% with lomustine alone; the median PFS was 4 months in the combination treatment, 3 months with bevacizumab alone, and 2 months in lomustine arm. And so, the combination treatment with lomustine and bevacizumab showed more effective than the both drugs given as single agent. Noteworthy, also in that study, such as the combination treatment of bevacizumab and fotemustine, the dosage of the cytotoxic drug was initially too toxic due to important thrombocytopenia. On the other side, the pharmacologic profile of fotemustine can be more beneficial than that of lomustine in the treatment of gliomas. In fact, fotemustine is more lipophilic than lomustine and so can facilitate fotemustine passage through the blood-brain barrier [3]; moreover, fotemustine demonstrated greater diffusion in neuronal cells and glia than lomustine and carmustine [31]. Liver and kidney toxicity due to fotemustine treatment are moderate and less frequent when compared with other drugs belonging to the group of nitrosourea family [5]. Moreover, preclinical data also indicate that fotemustine is more active than other nitrosoureas in mouse and human cell lines of glioma [3]. Furthermore, in a large, phase III study [32], median survival of only 9.8 months and median PFS of only 82 days (about 3 months), with an incidence of grades 3-4 thrombocytopenia of 22%, grades 3-4 lymphopenia of 8%, and grades 3-4 neutropenia of 3% were reported in 64 patients with recurrent glioblastoma treated with lomustine (110 mg/m^2^). On the contrary, Addeo et al. [20] reported an incidence of grades 3-4 thrombocytopenia of only 7% and grades 3-4 leukopenia of 3% with fotemustine treatment. Moreover, although it is not methodologically correct to compare different studies, these data seem to suggest that the combination treatment with bevacizumab and fotemustine may be more active and tolerable than lomustine in patients with high grade gliomas.

## 6. Conclusions

Fotemustine is a third-generation nitrosourea with an ideal pharmacological profile to treat primitive brain tumors showing efficacy in all types of glioma. Although no prospective and randomized studies were performed about the efficacy of fotemustine in malignant gliomas, results from numerous retrospective and prospective single-arm phase II clinical trials demonstrated its activity both as single agent and in combination with other cytotoxic drugs or new targeted drugs such as bevacizumab.

## Figures and Tables

**Table 1:** The most important studies on fotemustine treatment before temozolomide era.

Authors	PTS	Median age	Median KPS	Types of gliomas	Prior treatment	FTM schedule	Line of FTM treatment	DCR	PFS	OS	Grades 3-4 toxicity
Frenay et al., 1991 [8]	38	49 (18–65)	70 (50–100%)	GBM (53%), AA (24%), others (23%)	Surgery^§^ (71%), RT (100%), CT (26%)	Standard*	1st and 2nd	73% (PR 26%, SD 47%)	21–32.7 wks	40–42 wks	Thrombocytopenia 23%; leukopenia 17%; nausea 8.6%

Ozkan et al., 2004 [9]	27	46 (23–70)	NA	GBM (63%), AA (37%)	Surgery^§^ (100%), RT (100%)	FTM 100 mg/m^2^ every 3 weeks	1st	NA	8 ms	11 ms	Thrombocytopenia 7%

Fazeny-Dörner et al., 2003 [10]	31	50 (23–65)	70 (NA)	GBM (100%)	Surgery^§^ (100%), RT (100%), CT (100%)	FTM 100 mg/m^2^ every + D 200 mg/m^2^ every 3 wks	2nd	55% (PR 3%, SD 52%)	17 wks	45 wks	Thrombocytopenia 10%; leukopenia 3%

Boiardi et al., 2001 [11]	16	54 (20–63)	80 (70–100)	GBM (69%), AA (25%), AOA (6%)	Surgery^§^ (94%), RT (100%), CT (94%)	FTM 125 mg/m^2^ day 4 + PCZ 300 mg/day for 4 days, every 4 weeks	2nd	50% (PR 6%, SD 44%)	2.6 ms	9.7 ms	Thrombocytopenia 17%; leukopenia 17%; anemia 33%

Frenay et al., 2000 [12]	33	58 (20–73)	NA	GBM (100%)	None	FTM 100 mg/m^2^ day 1, CDDP 33 mg/m^2^ days 1–3, VP16 75 mg/m^2^ days 1–3, monthly	1st	79% (CR 3%, PR 24%, SD 52%)	NA	10 ms	Thrombocytopenia or leukopenia 36%

FTM: fotemustine; KPS: Karnofsky performance status; RT: radiation therapy; CT: chemotherapy; PTS: patients; PFS: progression-free survival; OS: overall survival; DCR: disease control rate; GBM: glioblastoma; AA: anaplastic astrocytoma; AOA: anaplastic oligoastrocytoma; PCZ: procarbazine; CDDP: cisplatin; VP16: etoposide; D: dacarbazine; wk: week; ms: months, CR: complete response; SD: stable disease; PR: partial response; NA: not available.

*Standard: FTM 100 mg/m^2^ every week for 3 consecutive weeks followed by a 5-week rest period, subsequently, an infusion every 3 weeks; ^§^Surgery: complete or partial resection.

**Table 2:** The most important studies on fotemustine treatment during temozolomide era.

Authors	PTS	Median age	Median KPS	Types of gliomas	Prior treatment	FTM schedule	Line of FTM treatment	DCR	PFS	OS	Grades 3-4 toxicity
Scoccianti et al., 2008 [17]	27	56	80 (70–100)	GBM	Surgery^§^(93%), RT + TMZ (100%)	Standard*	2nd	48% (PR 30%, SD 18%)	5.7 ms	9.1 ms	Thrombocytopenia 11%; leukopenia 4%

Brandes et al., 2009 [18]	43	51 (34–68)	90 (70–100)	GBM	Surgery^§^ (93%), RT + TMZ (100%)	Standard*	2nd	42% (PR 7%, SD 35%)	1.7 ms	6 ms	Thrombocytopenia 15%; leukopenia 7%; nausea 5%; lymphopenia 10%; hypertransaminasemia 9%

Fabrini et al., 2009 [19]	50	56 (30–76)	90 (70–100)	GBM	Surgery^§^ (94%), RT + TMZ (100%)	Standard*	2nd	62% (CR 2%, PR 16%, SD 44%)	6.1 ms	8.1 ms	Thrombocytopenia 8%; leukopenia 10%; nausea 2%; hypertransaminasemia 2%

Addeo et al., 2011 [20]	40	53 (30–75)	90 (70–100)	GBM	Surgery^§^ (82%), RT + TMZ (100%)	FTM 80 mg/m^2^ every 2 wks for 5 cycles followed, after a 5-week rest period, by FTM 80 mg/m^2^ every 4 wks	2nd	65% (CR 2%, PR 23%, SD 40%)	6.7 ms	11 ms	Thrombocytopenia 7%; leukopenia 3%; Nausea 12%; hypertransaminasemia 10%

Fabi et al., 2010 [21]	40	57 (26–80)	80 (60–100)	GBM (75%), AA (15%), AOD (10%)	Surgery^§^ (95%), RT (92%). TMZ (100%)	FTM 60 mg/m^2^ weekly for 3 cycles followed, after a 5-wk rest period, by FTM 75 mg/m^2^ every 3 wks	2nd and 3rd	52% (PR 20%, SD 32%)	3 ms	6 ms	Thrombocytopenia 7%; leukopenia 10%; nausea 2%; hypertransaminasemia 2%

Santoni et al., 2013 [22]	65	70 (≥65)	>70	GBM	Surgery^§^ (82%), RT + TMZ (100%)	Standard*	2nd	43% (CR 1%, PR 18%, SD 28%)	4.2 ms	7.1 ms	Thrombocytopenia 15%; leukopenia 9%; hypertransaminasemia 5%; anemia 3%

Silvani et al., 2008 [23]	54	53 (26–68)	80 (60–100)	GBM	Surgery^§^ (100%), RT (100%), TMZ (100%)	PCZ 450 mg days 1-2, 300 mg day 3; FTM 110 mg/m^2^ day 3 every 5 weeks	2nd and 3rd	65% (PR 11%, SD 54%)	19.3 wks	28.7 wks	Thrombocytopenia 1%; leukopenia 3%; hypertransaminasemia 1%

Soffietti et al., 2012 [29]	32	46	NA	Grade III glioma	Surgery^§^ (100%), RT + TMZ (100%)	FTM 75 mg/m^2^ days 1, 8, BV 10 mg/Kg days 1, 15 followed, after a 3 wk rest period, by FTM 75 mg/m^2^ + BV 10 mg/Kg day 1 every 3 wks	2nd	94% (CR 12%, PR 38%, SD 44%)	5 ms	8.6 ms	16% myelotoxicity

Soffietti et al., 2014 [28]	54	57	80 (60–100)	GBM	Surgery^§^ (92%), RT (100%), TMZ (100%)	FTM 75 mg/m^2^ days 1, 8, BV 10 mg/Kg days 1, 15 followed, after a 3 wk rest period, by FTM 75 mg/m^2^ + BV 10 mg/Kg day 1 every 3 wks	2nd	89% (CR 4%, PR 48%, SD 37%)	5.2 ms	9.1 ms	Thrombocytopenia 9%; leukopenia 13%; hypertension 2%; PE and DVT 4%

FTM: fotemustine; KPS: Karnofsky performance status; RT: radiation therapy; CT: chemotherapy; PTS: patients; PFS: progression-free survival; OS: overall survival; DCR: disease control rate; GBM: glioblastoma; AA: anaplastic astrocytoma; AOA: anaplastic oligoastrocytoma; PCZ: procarbazine; CDDP: cisplatin; VP16: etoposide; BV: bevacizumab; PE: pulmonary embolism; DVT: deep venous thrombosis; wk: week; ms: months; CR: complete response; SD: stable disease; PR: partial response; NA: not available.

*Standard: FTM 100 mg/m^2^ every week for 3 consecutive weeks followed by a 5-week rest period, subsequently, an infusion every 3 weeks; ^§^Surgery: complete or partial resection.
